# Structural basis underlying viral hijacking of a histone chaperone complex

**DOI:** 10.1038/ncomms12707

**Published:** 2016-09-01

**Authors:** Hongda Huang, Zhong Deng, Olga Vladimirova, Andreas Wiedmer, Fang Lu, Paul M. Lieberman, Dinshaw J. Patel

**Affiliations:** 1 Structural Biology Program, Memorial Sloan-Kettering Cancer Center, New York, New York 10065, USA; 2 Gene Expression and Regulation Program, The Wistar Institute, Philadelphia, Pennsylvania 19104, USA

## Abstract

The histone H3.3 chaperone DAXX is implicated in formation of heterochromatin and transcription silencing, especially for newly infecting DNA virus genomes entering the nucleus. Epstein-Barr virus (EBV) can efficiently establish stable latent infection as a chromatinized episome in the nucleus of infected cells. The EBV tegument BNRF1 is a DAXX-interacting protein required for the establishment of selective viral gene expression during latency. Here we report the structure of BNRF1 DAXX-interaction domain (DID) in complex with DAXX histone-binding domain (HBD) and histones H3.3-H4. BNRF1 DID contacts DAXX HBD and histones through non-conserved loops. The BNRF1-DAXX interface is responsible for BNRF1 localization to PML-nuclear bodies typically associated with host-antiviral resistance and transcriptional repression. Paradoxically, the interface is also required for selective transcription activation of viral latent cycle genes required for driving B-cell proliferation. These findings reveal molecular details of virus reprogramming of an antiviral histone chaperone to promote viral latency and cellular immortalization.

## Abstract (web summary)

The Epstein-Barr virus tegument protein BNRF1 is required for the establishment of selective viral gene expression during latency and interacts with the histone chaperone DAXX. Here the authors provide structural insight into how BNRF1 hijacks the DAXX-histone H3.3-H4 complex.

## Graphical abstract

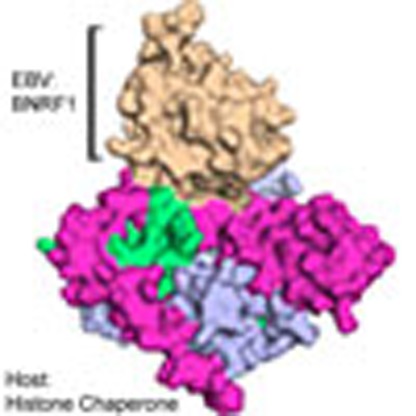

Chromatin assembly is a dynamic process that regulates all aspects of DNA biology, including transcription, replication and repair^1^. Histone chaperones perform a central function in the chromatin assembly process^2^,^3^,^4^,^5^. Several different classes of histone chaperones have been found to load specific histone variants to form distinct types of chromatin structures^6^,^7^. DAXX (death-domain associated protein-6) is a histone H3.3 chaperone that functions with the co-chaperone ATRX (ATP-dependent chromatin remodeller α-thalassaemia X-linked mental retardation protein) to assemble repressive heterochromatin at GC-rich repetitive elements, such as telomeres, pericentromeric regions and endogenous retrotransposons^8^,^9^,^10^,^11^,^12^. Chromatin organization can also function as a mechanism of resistance to viral infection^13^,^14^,^15^. Many viruses have acquired mechanisms to subvert the antiviral chromatin assembly factors, including DAXX and ATRX^16^. Importantly, each virus targets these factors in distinct ways that drive different infection strategies.

Epstein-Barr virus (EBV) is a human tumour virus that establishes long-term latent infection as a chromatinized episome^14^. During latent infection, EBV expresses a limited number of viral genes essential for viral genome persistence and host cell survival. The establishment of latency requires viral tegument proteins that are delivered with the viral DNA^17^. BNRF1 is an EBV-encoded tegument protein that is essential for transcription of viral genes during the earliest stages of viral infection^18^,^19^. BNRF1 is a member of a family of proteins with homology to the FGARAT enzyme involved in the *de novo* purine nucleotide biosynthesis^14^. Previously, we found that BNRF1 binds to DAXX to promote selective viral gene expression^18^,^19^. We now report the structural basis of how BNRF1 interacts with DAXX in a stable quaternary complex with histones H3.3 and H4. BNRF1 DAXX-interaction domain (DID) uses four extended loop domains not conserved in its cellular or viral orthologs, to contact all the components including DAXX histone-binding domain (HBD), H3.3 and H4 of the complex. We further show that the BNRF1-DAXX interface is responsible for BNRF1 localization to PML-nuclear bodies that are involved in host-antiviral resistance and transcriptional repression, and is also required for the selective transcription activation of viral latent cycle genes required for driving B-cell proliferation. These data reveal how the virus protein BNRF1 hijacks the cellular antiviral histone chaperone DAXX to promote viral latency and cellular immortalization.

## Results

### Structure of BRNF1 DID—DAXX HBD—H3.3-H4 complex

**Figure 1:**
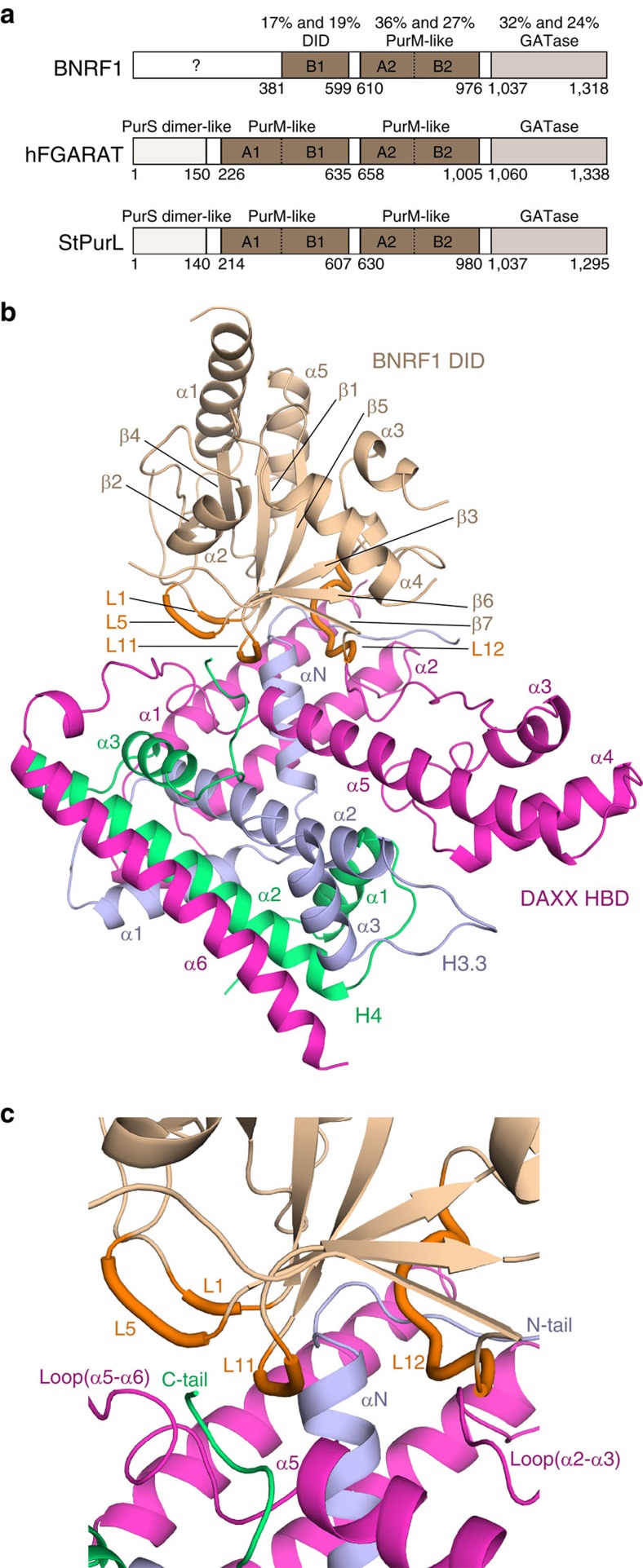
Crystal structure of the quaternary BNRF1 DID—DAXX HBD—H3.3-H4 complex. (**a**) Domain structure of the BNRF1, human FGARAT (hFGARAT) and *Salmonella typhimurium* FGARAT (StPurL). The numbers show the sequence identities of BNRF1 with hFGARAT and StPurL, respectively. (**b**) Representative view of the overall structure of the quaternary BNRF1 DID—DAXX HBD—H3.3-H4 complex highlighting the L1, L5, L11 and L12 loops interacting with DAXX HBD—H3.3-H4 complex. (**c**) An expanded view of the intermolecular interface in the quaternary complex.

BNRF1 is a member of a family of γ-herpesvirus tegument proteins that share significant homology in their C-terminal domains to the cellular purine biosynthesis enzyme FGARAT (FGARAT) (Fig. 1a). The N-terminal domains of this family have acquired distinct capacities to bind various components of the host antiviral machinery, including SP100, DAXX and RIG-I (ref. 20). BNRF1 is unique among these viral proteins for its capacity to bind DAXX through an N-terminal subdomain, termed the DID (aa 360–600) (Fig. 1a)^18^,^19^. On the basis of earlier findings by our groups and others revealing the structure of the ternary complex of the HBD of chaperone DAXX bound to the histone H3.3-H4 dimer^21^,^22^, and biochemical evidence for quaternary complex formation between this ternary complex and the DID of BNRF1 (refs 18, 19), we had successfully reconstituted and crystallized the quaternary complex of the BNRF1 DID (fragment 381–599; Fig. 1a) with the chaperone DAXX HBD and H3.3-H4 dimer. The structure of the quaternary complex shows that the BNRF1 DID folds into a globular α/β domain and is positioned on top of the DAXX HBD—H3.3-H4 complex (Fig. 1b,c) with X-ray structural statistics listed in Table 1 and a portion of the electron density map shown in Supplementary Fig. 1a.

BNRF1 DID has seven anti-parallel β-strands (β1 to β7) forming a β-sheet core fold, which is further surrounded on both sides by five α-helices (α1–α5) (Fig. 1b). A DALI search involving the fold of the BNRF1 DID alone based on the quaternary complex revealed that the overall fold is highly similar to the structure of the B-lobe of the first PurM-like domain of the *Salmonella typhimurium* FGARAT (StFGARAT or StPurL) protein^23^ (Fig. 1a), with an r.m.s.d. of 2.1 Å (Supplementary Fig. 1b). It is also apparent that there are extensive local differences spread throughout the entire BNRF1 DID fold, especially in that the BNRF1 DID has an unique and extremely long loop (L12 loop connecting β6 and β7) (Supplementary Fig. 1b), which is important for binding to DAXX HBD—H3.3-H4 (see below). The DAXX HBD—H3.3-H4 portion of the structure of the quaternary BNRF1 DID—DAXX HBD—H3.3-H4 complex, is similar to our previous structure of the ternary DAXX HBD—H3.3-H4 complex^21^, with small local differences in the binding interface for BNRF1 DID and with a small r.m.s.d. of 0.7 Å (Fig. 1b and Supplementary Fig. 1c).

### Intermolecular contacts in the quaternary complex

The BNRF1 DID uses four loops (L1, L5, L11 and L12) to capture the chaperone DAXX HBD—H3.3-H4 complex through contacts to each component of this complex (Fig. 1b,c). Briefly, BNRF1 DID binds to DAXX HBD in the regions of the latter's C-terminal tip of the α5 helix, the loop connecting the α5 and α6 helices and the loop between the α2 and α3 helices. It also binds to histone H3.3 in the regions of the loop before the αN helix and the base of the αN helix (residues 40–52), and binds to histone H4 in its C-terminal tail (residues 99–102) (Fig. 1c).

**Figure 2:**
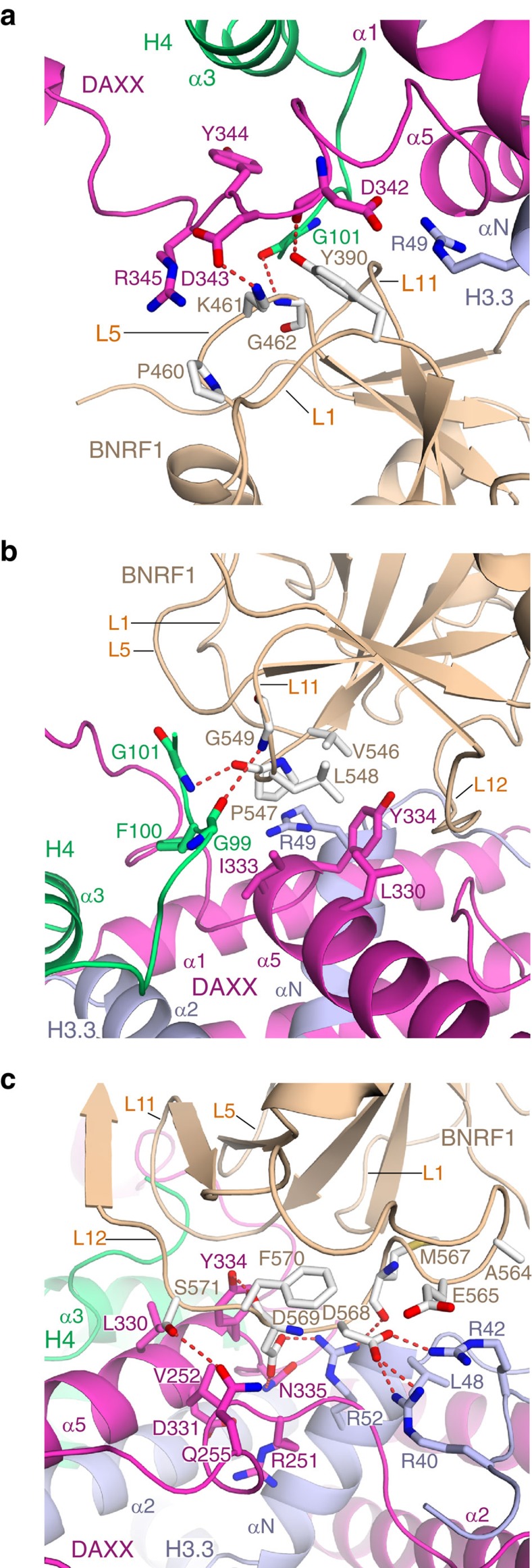
Details of intermolecular interactions of the quartenary complex. (**a**–**c**) Each panel highlights the details of intermolecular interactions of the BNRF1 DID with the DAXX HBD—H3.3-H4 complex. The interactions of the L1 and L5 loops (**a**), the L11 loop (**b**) and of the L12 loop (**c**) of the BNRF1 DID with the DAXX HBD—H3.3-H4 complex.

The detailed interactions amongst BNRF1 DID and DAXX HBD—H3.3-H4 are shown in the Fig. 2a–c. A single-residue Tyr390 from L1 loop and three residues, Pro460, Lys461 and Gly462 from L5 loop of BNRF1 DID interact with the residues Asp342, Asp343, Tyr344 and Arg345 in the loop between α5 and α6 of DAXX HBD, with the Arg49 of H3.3 and with the H4 C-terminal tail (Fig. 2a). These intermolecular interactions include hydrogen bonds and extensive van der Waals contacts.

The residues Pro547 and Gly549 from the L11 loop of BNRF1 DID form hydrogen bonds with Gly101 and Gly99 of H4 tail, respectively (Fig. 2b). Moreover, the Leu548 of BNRF1 DID inserts into a hydrophobic pocket formed by residues Leu330, Ile333 and Tyr334 in the tip of the α5 helix of DAXX HBD (Fig. 2b).

The acidic residues Asp568 and Asp569 from L12 loop of BNRF1 DID form extensive hydrogen bonds and electrostatic interactions with the basic residues Arg40, Arg42 and Arg52 of H3.3, and form hydrogen bonds with Tyr334 and Asn335 of DAXX HBD (Fig. 2c). In addition, interactions of the L12 loop of BNRF1 DID with residues Arg251, Val252 and Gln255 in the loop between α2 and α3 helices of DAXX HBD may also contribute to binding.

### Interfacial mutations influence the integrity of the complex

**Figure 3:**
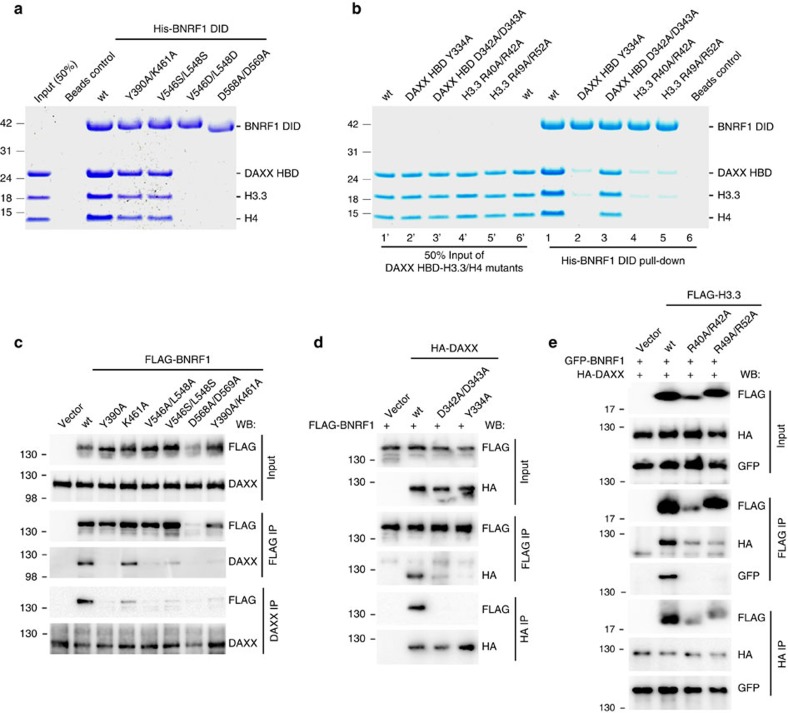
Impact of interfacial mutants on the BNRF1-DAXX-H3.3-H4 complex *in vitro* and in living cells. (**a**) *In vitro* pulldown of the BNRF1 DID wild-type (wt) and mutants with the DAXX HBD-H3.3-H4 complex. (**b**) *In vitro* pulldown of the BNRF1 DID wt with mutants of the DAXX-H3.3-H4 complex. The purifications of the mutants of the DAXX HBD-H3.3-H4 complex are shown in the Supplementary Fig. 2a,b. (**c**) IP-Western analysis of HEK293 cells transfected with expression plasmids for FLAG-BNRF1 wt or mutants, as indicated above each lane. Protein complexes were isolated by FLAG- or DAXX-IP and assayed by WB with either FLAG or DAXX antibody, as indicated. Input (10%) for FLAG and DAXX is shown in top panels. Molecular weight markers are indicated in kDa. (**d**) Same as in **c**, but HA-DAXX, or HA-DAXX mutants (as indicated) were co-transfected with FLAG-BNRF1 wt and subject to IP with either FLAG beads or HA antibody followed by WB with HA or FLAG antibodies, as indicated. (**e**) Same as in **c**, but FLAG-H3.3 or H3.3 mutants (as indicated) were co-transfected with GFP-BNRF1 and HA-DAXX, and then subject to IP with either FLAG beads or HA antibody, and assayed by WB with FLAG, HA or GFP antibody, as indicated. Gel source data, please see Supplementary Fig. 7.

To validate the intermolecular interactions identified by the X-ray structure of the quaternary complex, we tested mutations in the BNRF1 interface for their effect on complex formation *in vitro* (Fig. 3a, b and Supplementary Fig. 2a, b), as well as in living cells (Fig. 3c–e and Supplementary Fig. 2c–e). We used *in vitro* pulldown assays to validate the observed intermolecular interactions between BNRF1 DID and the chaperone DAXX HBD-H3.3-H4 complex. The dual mutations V546D/L548D and D568A/D569A on BRNF1 DID (Fig. 3a), as well as R40A/R42A and R49A/R52A on H3.3 and Y334A on DAXX HBD (Fig. 3b) abrogate quaternary complex formation. Our data also show that dual mutations Y390A/K461A and V546S/L548S on BNRF1 DID, and D342A/D343A on DAXX HBD partially diminish, but do not eliminate complex formation *in vitro*.

We next assayed by immunoprecipitation (IP) the ability of FLAG-tagged BNRF1 or site-directed mutants for interaction with DAXX in IPs with either FLAG or DAXX antibody after transfection into 293HEK cells. Substitution mutations in BNRF1 containing either Y390A, V546A/L548A, V546S/L548S, D568A/D569A or Y390A/K461A severely disrupted BNRF1-DAXX interaction, with the D568A/D569A mutant causing instability in BNRF1 (Fig. 3c and Supplementary Fig. 2c). Mutant K461A had a modest effect on BNRF1-DAXX interaction. Similar results were observed when HA-DAXX and FLAG-BNRF1 wt or mutants were co-transfected into 293HEK cells and assayed in IPs with either FLAG or HA antibody (Supplementary Fig. 2d). Mutations in the DAXX interface at positions D342A/D343A or Y334A disrupted the interaction with BNRF1 in both FLAG-BNRF1 and HA-DAXX IPs (Fig. 3d). The contribution of histone H3.3 was assayed by co-transfection of FLAG-H3.3, GFP-BNRF1 and HA-DAXX into 293HEK cells. Mutations in histone H3.3 at positions R40A/R42A or R49A/R52A disrupted the interaction with GFP-BNRF1 in FLAG-H3.3 IP experiments (Fig. 3e). These mutations reduced, but did not eliminate their interaction with HA-DAXX either in the presence or absence of GFP-BNRF1, with R40A/R42A causing instability in histone H3.3 (Fig. 3e and Supplementary Fig. 2e). These findings with full-length BRNF1 and DAXX in living cells substantiate the importance of the interfacial contacts determined by the structure for maintaining a quaternary complex in living cells.

### Mutants on BNRF1 reduce its co-localization with DAXX

**Figure 4:**
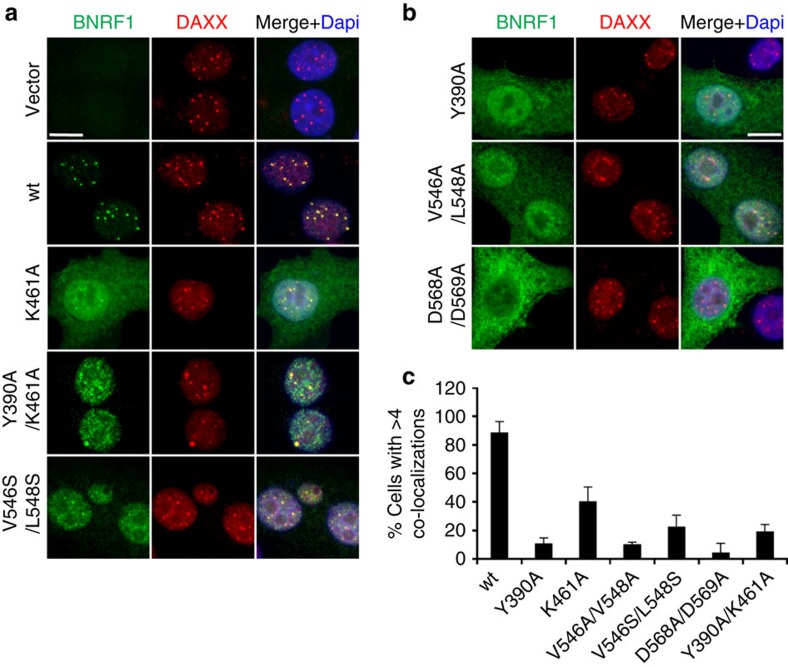
Interfacial mutants on BNRF1 exhibit reduced subnuclear co-localization with DAXX. (**a**,**b**) HEP2 cells were transfected with FLAG-BNRF1 wt or mutants (as indicated) for 24 h and then assayed by IF with antibodies to FLAG (green), DAXX (red) and Dapi (blue) in Merge image. Scale bar, 20 μm. (**c**) Quantification of cells with BNRF1 foci co-localizing with DAXX foci. Cells were scored positive only if >4 BNRF1 foci co-localized with DAXX foci. The bar graph shows means±s.d. derived from quantification of over 100 nuclei for each transfection from multiple independent IF assays (*n*=4).

We next assayed the importance of the BNRF1-DAXX interface on BNRF1 subcellular localization (Fig. 4a–c). BNRF1 wt co-localized strongly with DAXX at punctate subnuclear structures previously shown to be PML-NBs (promyelocytic leukemia-nuclear bodies). BNRF1 mutations at K461A, Y390A/K461A and V546S/L548S reduced, but did not eliminate BNRF1 co-localization at PML-NBs (Fig. 4a). Mutations at Y390A, V546A/L548A and D568A/569A showed a diffuse cytoplasmic pattern and severely reduced the frequency of BNRF1 co-localization with DAXX at PML-NBs (Fig. 4b). Biochemical fractionation studies, which do not involve the wash conditions used for microscopy, indicated that BNRF1 and DAXX have both cytoplasmic and nuclear distributions, and that mutations in the DAXX interface did not alter this overall distribution (Supplementary Fig. 3). These findings indicate that BNRF1 interfacial mutants were not compromised for nuclear localization, but BNRF1 localization to PML-NBs depends on its interaction with DAXX.

### Functional significance of BNRF1-DAXX interaction

**Figure 5:**
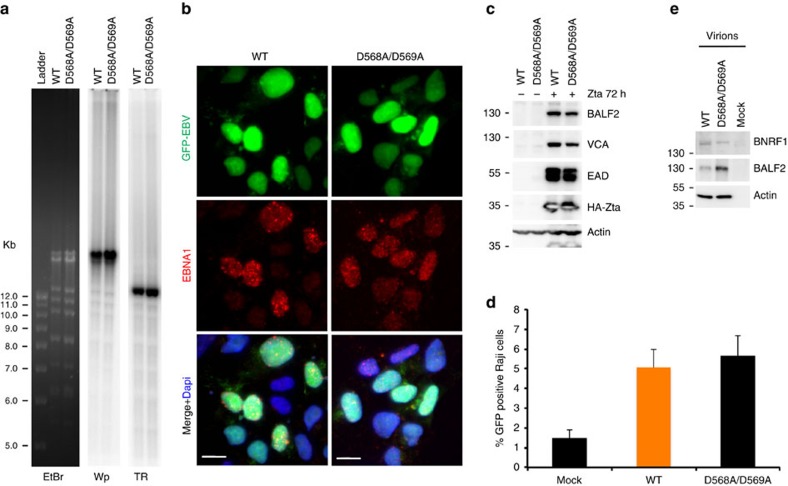
Generation of the recombinant EBV-GFP BNRF1 D568A/D569A bacmid and virus. (**a**) BACEBV-GFPwt and BNRF1D568A/D569A mutant was digested with *Eco*RI, fractionated on 0.8% agarose gel, and stained with ethidium bromide (EtBr) followed by Southern blots with ^32^P-labelled probes specific for Wp or TR regions. (**b**) Immunofluorescence analysis for EBNA1 (red) in BACEBV-GFPwt and BNRF1D568A/D569A HEK-293T virus production cell lines. GFP expression levels (green) were monitored and Dapi (blue) was shown in Merge image. Scale bar, 10 μm. (**c**) Virus production cells shown in **b** were either mock transfected or co-transfected with expression vectors for HA-tagged Zta and BALF4 for 72 h. Viral reactivation was examined by WB with BALF2, VCA, EAD, HA or actin antibody, as indicated. (**d**) Raji cells were mock treated or superinfected with recombinant EBV wt (orange) or BNRF1 D568A/D569A genomes at a MOI of 30 for 4 days in the presence of 100 ng ml^−1^ TPA and then assayed for Raji cell infection by FACS analysis of GFP-positive cells. The bar graph represents means±s.d. (*n*=3). (**e**) Virions from HEK-293T mock cells or recombinant wt or D568A/D569A virus production cells were assayed by WB with antibodies to BNRF1, BALF2 or actin. FACS, fluorescence-activated cell sorting; MOI, multiplicity of infection; TPA, tetradecanoyl phorbol acetate.

**Figure 6:**
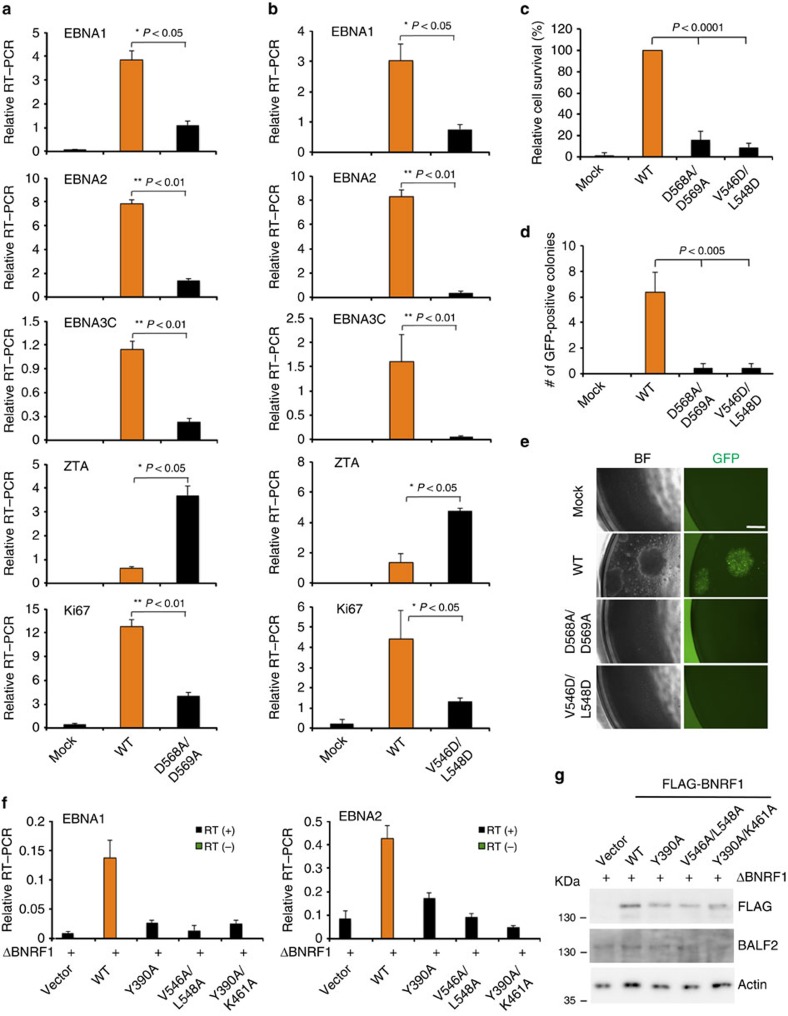
BNRF1-DAXX interaction is essential for EBV latent cycle gene expression during primary infection and B-cell immortalization. (**a**) Primary human B-lymphocytes were mock treated or infected with recombinant EBV wt (orange) or BNRF1 D568A/D569A genomes at a MOI of 30 for 1 week and then assayed by RT–PCR for EBV latency-associated genes EBNA1, EBNA2, EBNA3C or lytic immediate early gene ZTA, or cellular proliferation marker Ki67. The bar graph represents means±s.d. from at least three independent infection experiments. *P* value was calculated by two-tailed *t*-test. (**b**) The same as in **a**, except that BNRF1 V546D/L548D mutant virus was used to infect primary B cells. (**c**) Resazurin assay measuring the relative cell survival for B-cells infected with mock, EBV wt, D568A/D569A or V546D/L548D recombinant virus at 4 weeks post infection. Data represent means±s.d (*n*=3), two-tailed *t*-test. (**d**) Colony formation assay for primary B cells infected with mock, EBV wt, D568A/D569A or V546D/L548D recombinant virus at 3 weeks post infection. Data represent means±s.d (*n*=3), two-tailed *t*-test. (**e**) Representative images of the B-cell clonal proliferation used for quantification, as shown in **d**. BF indicates bright-field imaging, and scale bar, 200 μm. (**f**) Primary B-lymphocytes were infected at a MOI of 10 with recombinant EBV virus generated by trans-complementation of the BNRF1 deletion mutant (ΔBNRF1) with either empty vector, FLAG-BNRF1 wt, Y390A, V546A/L548A or Y390A/K461A. EBNA1 and EBNA2 expression was assayed by quantitative real-time PCR at 1 week post infection. Data represent means±s.d from two independent experiments with triplicates. No RT controls shown in green. (**g**) WB for virions generated by trans-complementation with FLAG-BNRF1 wt or mutants, as indicated, and probed with FLAG, BLAF2 or actin. MOI, multiplicity of infection; RT–PCR, PCR with reverse transcription.

We next tested the functional significance of the BNRF-DAXX interaction by generating recombinant EBV genomes with either D568A/D569A (Figs 5 and 6) or V546D/L548D (Fig. 6 and Supplementary Fig. 4), or by trans-complementation of a BNRF1 deletion virus with either BNRF1 wt, Y390A, V546A/L548A or Y390A/K461A (Fig. 6). Viruses generated from these different sources were validated for integrity by restriction digest and Southern blot for terminal and internal repeat sequences (Fig. 5a and Supplementary Fig. 4a), as well as for viral gene expression by IF and western blot (WB) (Fig. 5b, c and Supplementary Figs 4b,c and 5a). Virus produced from these cells was normalized for viral DNA copies by quantitative PCR and formation of GFP-positive Raji cells after infection (Fig. 5d and Supplementary Fig. 4d). Virion proteins were further examined by WB, and we found that both wt and BNRF1 mutant viruses contain comparable level of viral BNRF1, BALF2 and cellular protein Actin, all of which have previously been identified in proteomic studies of EBV virions^24^ (Figs 5e and 6g and Supplementary Fig. 4e). We did not detect any western signals for these proteins in mock preparations from HEK-293T cells, indicating that the virus preparations are specific for the virus production cells.

To determine the functional significance of BNRF1-DAXX interaction, quantitative PCR normalized units of wt and BNFR1 mutant viruses were used to infect primary human B-lymphocytes (Fig. 6). Viral gene expression was assayed by quantitative real-time PCR at 1 week post infection. We found that all BNRF1 mutations that compromised DAXX binding were defective for latency-associated transcription (for example, EBNA1, EBNA2, EBNA3C mRNA) and cellular proliferation marker Ki67 relative to wt BNRF1 virus (Fig. 6a,b,f and Supplementary Fig. 5b). Interestingly, recombinant virus-containing D568A/569A and V546D/L548D mutations showed elevated levels of ZTA immediate early gene transcripts, suggesting that BNRF1-DAXX interaction is also required for suppression of early lytic cycle gene activity. The similar pattern of changes in viral gene expression was observed at 72 h after viral infection (Supplementary Fig. 5c). Moreover, neither D568A/L569A nor V546D/L548D were capable of inducing lymphoblast proliferation and colony formation similar to wt EBV, indicating that the BNRF1-DAXX interaction is essential for B-cell immortalization (Fig. 6c–e).

## Discussion

Our studies indicate that BRNF1 DID contacts all three components of the chaperone complex, namely DAXX HBD, H3.3 and H4 (Fig. 1b). A network of intermolecular hydrogen bonds, electrostatic and hydrophobic interactions mediate recognition at the interface in the quaternary complex (Fig. 2). The *in vitro* and cell-based mutational studies (Fig. 3) emphasize the importance of the interactions of the acidic L12 loop (Asp568 and Asp569) of BNRF1 DID with the basic regions (Arg40, Arg42, Arg49 and Arg52) of H3.3, given loss in binding on Ala substitution at these positions. Further, studies with the Y334A mutant emphasize that Tyr334 of DAXX HBD is also important for the binding with BNRF1 DID. The above intermolecular recognition elements in our structure of the quaternary BNRF1 DID—DAXX HBD—H3.3-H4 complex and their response to interfacial mutations provide definitive insights into the structural basis underlying how BNRF1 interacts with the histone chaperone DAXX—H3.3-H4 complex.

Our work also reveals that BNRF1 interfacial mutants were not compromised for nuclear localization, but BNRF1 localization to PML-NBs depends on its interaction with DAXX (Fig. 4). Recombinant viruses harbouring either D568A/D569A or V546D/L548D mutations show defects in activating viral latent gene expression and driving B cell proliferation, suggesting that BNRF1 localization to PML-NBs mediated by BNRF1-DAXX interface is required for the establishment of viral latency program during primary infection (Fig. 6). Our analysis of these bacmids by restriction digest and Southern blot for terminal and internal repeat sequences showed that BNRF1 mutations did not alter the integrity of viral genome (Fig. 5). In addition, WB of viral reactivation from virus production cells revealed that recombinant viruses harbouring the BNRF1 mutations were induced at a comparable level of viral gene expression as the wild-type viruses. However, WB analysis of virion proteins indicated that BNRF1 mutant viruses D568A/D569A and V546D/L548D expressed increased levels of BALF2 and BNRF1, respectively relative to their wild-type viruses. While increased levels of BALF2 or BNRF1 in mutant virions are unlikely to lead to the reduction in viral latent gene expression during primary infection, we cannot exclude the possibility that BNRF1 mutations alter the composition and assembly of virions, which in turn affect infectivity, viral gene expression, and cellular proliferation. Future experiments including electron microscopy of virions will be necessary for validating that BNRF1 mutations modulate viral gene expression by lack of interaction with DAXX, but not by the structural changes in mutant virions.

While our data clearly demonstrated that mutations in BNRF1/DAXX interface disrupt BNRF1 and DAXX interactions both *in vitro* and *in vivo*, the underlying mechanisms how BNRF1 mutations compromise viral infection into primary B cells remains unclear. Our earlier studies suggested that BNRF1 activates early gene transcription through the disruption of ATRX-DAXX interaction and the establishment of a chromatin structure permissive for viral early gene activity^18^,^19^. To this end, future studies by ChIP analysis of ATRX/DAXX occupancy and chromatin assembly at viral genomes after primary infection with BNRF1 mutant or wild-type viruses will be necessary to elucidate how BNRF1-DAXX interactions contribute to the establishment of viral latency.

Notably, despite EBV and KSHV being in the same family of γ-herpesvirus, the BNRF1 of EBV interacts with the DAXX—H3.3-H4 complex, while its KSHV homologue ORF75 does not^20^. The sequence alignment of these two species reveals that the BNRF1 residues identified by our structure to be important for binding to the DAXX—H3.3-H4 complex are not conserved in KSHV ORF75 (Supplementary Fig. 6), thus explaining why BRNF1 but not KSHV ORF75 interacts with the DAXX—H3.3-H4 complex. Importantly, the BNRF1 interaction with DAXX—H3.3-H4 is essential for BNRF1 localization to PML-NBs and the selective activation of EBV latent, but not lytic cycle gene transcription. These data support the model that BNRF1 reprograms DAXX histone chaperone function to promote the exclusive expression of EBV latent cycle genes essential for EBV induced B-cell proliferation and immortalization.

## Methods

### Protein expression and purification

The cDNA of human DAXX HBD (178–389) was cloned into a modified RSFDuet-1 vector (Novagen), while the cDNA of human histones H3.3 and H4 were cloned into one pETDuet vector (Novagen). The DAXX HBD (178–389)—H3.3-H4 ternary complex was coexpressed and prepared in *Escherichia coli*^21^. The cDNA of EBV BNRF1 DID (381–599) was synthesized at GENEWIZ and was cloned into a modified RSFDuet-1 vector (Novagen), with an N-terminal His_6_-SUMO tag. The BNRF1 DID was expressed in BL21(DE3)-RIL cell strain (Stratagene). The expressed protein complex was first purified on nickel-sepharose affinity column (GE Healthcare). After removing the His_6_-SUMO tag by using Ulp1 (SUMO protease), the protein complex was further purified on HiLoad 16/600 Superdex 200 column (GE Healthcare). Equal moles of the BNRF1 DID protein and the DAXX HBD—H3.3-H4 ternary complex were mixed together and incubated on ice for 10 min (mins) to form the quaternary BNRF1 DID—DAXX HBD—H3.3-H4 complex, then the quaternary complex was purified with HiLoad 16/600 Superdex 200 column. The resulting quaternary complex was concentrated to 5.8 mg ml^−1^ in a buffer of 20 mM Tris pH 7.5 and 1 M NaCl.

### Crystallization

The quaternary BNRF1 DID—DAXX HBD—H3.3-H4 complex in a concentration of 5.8 mg ml^−1^ was crystallized in 0.1M MES pH 6.0, 0.8 M ammonium sulfate using sitting-drop vapour-diffusion method at 20 °C. All the crystals were soaked in a cryoprotectant made from mother liquor supplemented with 20% glycerol before flash freezing in liquid nitrogen.

### Structure determination

The data sets for the quaternary BNRF1 DID—DAXX HBD—H3.3-H4 complex were collected at 0.979 Å on 24-ID-C/E NE-CAT (Advanced Photo Source, Argonne National Laboratory). All the data sets were processed by using the HKL 2,000 program. The initial structure for the quaternary BNRF1 DID—DAXX HBD—H3.3-H4 complex was solved by molecular replacement in PHASER^25^ with the structure of the ternary DAXX HBD–H3.3-H4 complex (PDB 4H9N) as a search model and manually refined and built using Coot^26^. The final structure of this complex was refined to 3.60 Å resolution using PHENIX^27^. The Ramachandran plot was calculated with PHENIX and showed 93.8% favoured and 6.2% allowed. Table 1 summarizes the statistics for data collection and structural refinement.

### Cells for this study

Hep2, 293HEK, and 293T cells were obtained from ATCC and grown in Dulbecco's modified Eagle medium (DMEM) supplemented with 10% fetal bovine serum (FBS), 100U ml^−1^ penicillin-streptomycin. All these cell lines were validated to be free of mycoplasma contamination and were used for transfection experiments. Raji cells were validated for EBV genome integrity and grown in RPMI 1640 medium supplemented with 10% FBS, 100 U ml^−1^ penicillin-streptomycin. Purified human primary B cells were purchased from Path BioResource (Department of Pathology and Laboratory Medicine, University of Pennsylvania). Primary B cells isolated from various blood donors, including ND388, ND365, ND473, ND317, ND343 and ND422, were cultured in RPMI 1640 medium supplemented with 20% FBS, 10 mM Hepes, pH 7.2, 1 mM Sodium Pyruvate, 10 mM GlutaMAX (Gibco) and 100 U ml^−1^ penicillin-streptomycin. All cells were grown in a 5% CO_2_ incubator at 37 °C.

### Cloning and mutagenesis

pcDNA3-HA-DAXX was a generous gift from H.-M. Shih^28^, and FLAG-BNRF1 expression vectors were generated by PCR amplification and cloning into *Hind*III/*Sal*I sites of the p3xFLAG-Myc-CMV-24 Expression Vector (Sigma-Aldrich)^18^. An EGFP-BNRF1 expression plasmid was generated by PCR amplification and cloning into *Hind*III/*Sal*I sites of pEGFP-C3 vector (Clonetech). A FLAG-H3.3 expression vector was constructed by PCR amplification and cloning into *Hind*III/*Bam*HI sites of p3xFLAG-CMV vector (Sigma). FLAG-BNRF1 mutants (Y390A, K461A, V546A/L548A, V546S/L548S, D568A/D569A and Y390A/K461A), HA-DAXX mutants (D342A/D343A and Y334A), and FLAG-H3.3 mutants (R40A/R42A and R49A/R52A) were generated by QuickChange Site-directed Mutagenesis (Stratagene). All constructs were verified by sequencing and cloning details are available upon request.

### Co-Immunoprecipitation

Co-Immunoprecipitation (Co-IP) was performed in HEK293 cells transfected with indicated expression plasmids using Lipofectamine 2000 (Invitrogen). Cells were harvested 48 h post transfection, washed two times with cold PBS, and then lysed in cold lysis buffer (20 mM Tris, pH 8.0, 137 mM KCl, 1 mM EDTA, 1.5 mM MgCl_2_, 10% Glycerol, and 1% Triton X-100 supplemented with 1 mM DTT and 0.1% mammalian protease inhibitor cocktail mix) for 30 min on ice. Cell lysates were centrifuged at about 18,000*g* for 10 min, and the supernatants were precleared with Protein G Sepharose beads (GE Healthcare) for 60 min at 4 °C with rotation. One ml of precleared lysates (∼5 × 10^6^ cells) were immunoprecipitated with either FLAG agarose beads (Sigma) or mouse monoclonal anti-HA (12CA5, Roche) or rabbit anti-DAXX (Sigma) overnight at 4 °C with rotation. For HA and DAXX IP, the immuno-complex was collected with Protein G sepharose beads with rotating at 4 °C for 3 h. The beads were washed three times with BC300 or BC500 (500 mM KCl, 20 mM Tris-HCl, pH 8.0, 0.2 mM EDTA, 10% glycerol and 10 mM β-mercaptoethanol) for endogenous DAXX or for HA-DAXX, respectively, and then followed by once with BC100 at 4 °C. Pulled down proteins were eluted by either FLAG peptide or boiling with 2 × Laemmli buffer (100 mM Tris-HCl, pH 6.8, 4% SDS, 0.2% Bromophenol Blue, and 20% Glycerol), and were subject to SDS–polyacrylamide gel electrophoresis (8–16% Tris-Glycine gel, Invitrogen) and WB analysis.

### Indirect immunofluorescence

Indirect immunofluorescence (IF) was performed in Hep2 cells transfected with BNRF1 expression plasmids using Lipofectamine 2000 (Invitrogen). Transfected cells were reseeded in 24-well plates with coverslips at 6 h post transfection. At 24 h post transfection, cells were rinsed in 1 × PBS, fixed with 1% paraformaldehyde in 1 × PBS at room temperature for 15 min, and permeablized with 0.3% Triton-X 100 for another 15 min. Coverslips were blocked in PBG solution (0.2% cold water fish gelatin, 0.5% BSA in 1 × PBS) for 30 min at room temperature. Cells were then stained sequentially with mouse monoclonal anti-FLAG (F1804, Sigma, 1:10,000 dilution in PBG) followed by rabbit anti-DAXX (F7810, Sigma, 1:800 dilution in PBG) at room temperature for 1 h each. Secondary antibody stainings were performed at toom temperature for 1 h with Alexafluor488 goat anti-mouse antibody and Alexafluor594 goat anti-rabbit antibody (Invitrogen) at 1:800 dilution in PBG, respectively. Nuclei were counterstained with 0.1 mg ml^−1^ DAPI in PBG and slides were mounted with FluoroMount-G (SouthernBiotech). Images were captured with a 100 × objective lens on a Nikon E600 Upright microscope (Nikon Instruments, Inc., Melville, NY, USA) using ImagePro Plus software (Media Cybernetics, Silver Spring, MD) and Adobe Photoshop CS5 for image processing. Transfected cells with four or more BNRF1 foci co-localizing with DAXX foci were scored as positive for BNRF1 nuclear co-localization with DAXX.

### Subcellular fractionation assay

Hep2 cells were transfected in 6 cm plates with 2 μg expression plasmids for either empty FLAG vector, or FLAG-tagged BNRF1 wt or mutants using Lipofectamine 2000 (Invitrogen). Cells were harvested 48 h post transfection and subject to REAP nuclear/cytoplasmic fractionation^29^. Briefly, cells were washed twice with ice-cold PBS, and collected with PBS into 1.5 ml microcentrifuge tube. Cell pellets were triturated five times with 1 ml ice-cold 0.1% NP40-PBS using p1000 micropipette. An aliquot (200 μl) was removed into a fresh tube as the whole-cell extracts, and the rest of samples was centrifuged at max speed for 1 min at 4 °C with table top centrifuge. An aliquot (200 μl) of supernatants was removed into a fresh tube as the cytoplasmic fraction, and the remainder of supernatants was removed by aspiration. The pellet was resuspended in 1 ml ice-cold 0.1% NP40-PBS, and centrifuged at max speed for 1 min at 4 °C with table top centrifuge. The supernatant was removed, and the nuclear pellet was resuspended with 1x Laemmli buffer (200 μl) and solubilized by brief sonication. The resulting cytoplasmic and nuclear fractions, along with the whole-cell extracts were analysed by WB.

### EBV bacmids and virus production cells

Viruses were produced using chloramphenicol and hygromycin resistant bacmids containing the EBV genome and the gene coding for green fluorescence protein (GFP). 293/EBV-wt and ΔBNRF1 cells (gifts from H.J. Delecluse) are 293T cells transfected with the wild-type EBV bacmid or an EBV bacmid with the *BNRF1* gene deleted^17^,^30^. EBV bacmids with D568A/D569A and V546D/L548D mutations in BNRF1 were generated from recombinant B95-8 and M81 genome, respectively by recombineering using two-step markerless red recombination method^31^. The mutations were confirmed by sequencing and restriction enzyme analysis with *Eco*RI on 0.8% agarose gel. To further assure that the recombineering process did not alter the rest of the genome, the gel was transferred and assayed by Southern blot for the integrity of Wp and TR regions using ^32^P-labelled Wp- or TR-specific probes. The images were analysed by Phosphor-imager, visualized by Typhoon 9410 Imager (GE Healthcare), and analysed with ImageQuant 5.2 software (Molecular Dynamics).

BNRF1 wt and mutant production cells were generated by transfecting the bacmid DNA into 293T cells with Effectene transfection reagent (Qiagen) and selection with 100 μg ml^−1^ hygromycin for 3 weeks. 293/EBV-wt and 293/BNRF1 mutant cells were continuously grown in DMEM medium supplemented with 10% FBS and 100 μg ml^−1^ hygromycin. The populations of virus production cells were confirmed by visualization of GFP and EBNA1 protein by immunofluorescence as described above. Images were captured with a 60 × objective lens on a Nikon E600 Upright microscope using ImagePro Plus software and Adobe Photoshop CS5 for image processing.

### Virus production and titre measurements

EBV lytic virus production was performed in 293/EBV wt and BNRF1 mutant cells co-transfected in 15 cm plates with expression plasmids for BALF4 (3.5 μg) and HA-tagged BZLF1 (7 μg) using 30 μl of Lipofectamine 2000 (Invitrogen). For the production of BNRF1 complemented viruses, 293/ΔBNRF1 cells were co-transfected with expression plasmids for BALF4, BZLF1 and either empty FLAG vector or FLAG-tagged BNRF1 wt or mutants (5 μg). At 24 h post transfection, a small fraction of transfected cells were replated into six-well plate and assayed by WB for viral reactivation at 72 h post transfection. The rest of transfected cells were plated into two 15 cm plates in 35 ml culture medium and induced for 6 days before harvest for virus preparation. The media of virus production cells were harvested by centrifugation at about 2,000*g* for 10 min and filtered through 0.45 mm filters. The concentrated virus were prepared by ultracentrifugation of the filtered media over 5 ml layer of 10% sucrose in 1 × PBS at 100,000*g* for 1 h at 4 °C with a Sorvall WX 100 ultracentrifuge, resuspended in B cell growth medium with 1/100 volume of original medium, and stored at −80 °C.

Viral titres were assayed by real-time PCR detection of DNA copy number^19^. Viral DNA was extracted by treatment of virus-containing media with 10 U per 50 μl of DNase I (New England Biolabs) for 1 h at 37 °C, followed by 10 min heat inactivation at 70 °C in the presence of 5 mM EDTA. Samples were further treated with 0.1 mg ml^−1^ of proteinase K at 50 °C for 1 h, followed by 20 min of heat inactivation at 75 °C. The released viral DNA was measured by real-time PCR analysis, using a serial dilution series of Namalwa cell lysate as the standard curve, which contain two copies of integrated EBV genome per Namalwa cell. EBV genomes were detected using primers specific to EBER region (forward, 5′-GGACAGCCGTTGCCCTAGTGGTTT-3′; reverse, 5′-CGATAAGCTTAAAAATAGCGGACAAGCCG-3′). Infection into Raji cells and WB of the concentrated virus were further used to examine the expression and packaging of BNRF1 proteins in corresponding virions. For Raji cells infection, mock medium or medium containing the concentrated virus was added into wells with about 2 × 10^5^ Raji cells at a concentration of 30 viral DNA copies per cell in triplicates. A total of 100 ng ml^−1^ tetradecanoyl phorbol acetate was also added to each well to amplify the GFP signal. The viral infection was determined 4 days after infection by measuring the percentage of GFP-positive Raji cells by fluorescence-activated cell sorting analysis with an EPICS XL flow cytometer (Beckman Coulter). For WB of virions, the concentrated virus was lysed in 2 × laemmli buffer, and protein gel loading volumes were normalized according to viral titres to ensure equal amounts of virion protein in each well. Equal volume of mock 293T media was treated in the same procedure as viral production and the resulting concentrated sample was used as a negative control in the WB analysis.

### Western blotting and antibodies

Equal amounts of samples in 1 × Laemmli buffer were electrophoresed on an 8–16% Tris-Glycine gel (Life Technologies), transferred to nitrocellulose membrane, blotted with the indicated antibodies, and visualized by ECL plus kit (GE Healthcare) using LAS 3,000 imager (Fuji Film). The antibodies used in IF and WB were as followings: Rabbit polyclonal rabbit anti-DAXX (F7810, 1:1,000 dilution), mouse monoclonal anti-beta-Actin-Peroxidase (A33854, 1:2,000 dilution), mouse monoclonal anti-FLAG M2-Peroxidase (A8592, 1:2,000 dilution), and anti-FLAG M2 Affinity Gel (A2220) were purchased from Sigma-Aldrich. Mouse monoclonal anti-EAD (MAB8186, 1:500 dilution), anti-EBV VCA (MAB8184, 1:500 dilution), and rabbit polyclonal anti-Histone H3 (06-755, 1:1,000 dilution) were purchased from EMD Millipore. Rabbit polyclonal anti-GAPDH (ab9485, 1:1,000 dilution) was purchased from Abcam. Mouse monoclonal anti-EBNA1 (MCA2707, 1:600 dilution) was purchased from BioRad. Mouse monoclonal anti-HA (12CA5, 1:1,000 dilution) was from Roche. Rabbit anti-BNRF1 (1:100 dilution) and anti-BALF2 (1:200 dilution) were raised against the peptides from BNRF1 and BALF2, and custom produced by YenZym Antibodies, LLC.

### Viral infection assay

Primary B cells were infected with concentrated EBV recombinant viruses at a ratio of 30 viral DNA copies per cell (multiplicity of infection of 30). Infected cells were harvested at specified time points for downstream assays. For measuring virus gene expression in infected B cells, cells were collected at 72 h, or 1 week post infection, and total RNA was purified using Trizol reagent (Invitrogen) as manufacturer's instruction. The RNA samples were treated with DNase I for 45 min at 37 °C, followed by DNase I inactivation in the presence of EDTA at 65 °C for 5 min. cDNA was synthesized using the Super Script IV Reverse Transcriptase (Invitrogen). The resulting cDNA was then subject to real time PCR analysis with ABI Prism 7900 Sequence Detection System (Applied Biosystems). Relative PCR with reverse transcription was determined from at least three independent viral infections using ΔCT methods relative to internal control *GUSB* gene. Real-time PCR primers used are as followings: *EBNA1* (forward, 5′-GGTCGTGGACGTGGAGAAAA-3′; reverse, 5′-GGTGGAGACCCGGATGATG-3′), *EBNA2* (forward, 5′-GCTTAGCCAGTAACCCAGCACT-3′; reverse, 5′-TGCTTAGAAGGTTGTTGGCATG-3′), *EBNA3C* (forward, 5′-GCCGGGCTGTCAAGCA-3′; reverse, 5′-CCCACTATCGAGTATCAGGTTTGAT-3′), *ZTA* (forward, 5′-TCTGAACTAGAAATAAAGCGATACAAGAA-3′; reverse, 5′-TTGGGCACATCTGCTTCAAC-3′), *Ki67* (forward, 5′-ATGCAGACCCAGTGGACACC-3′; reverse, 5′-TGCTGCCGGTTAAGTTCTCT-3′), *GUSB* (forward, 5′-CGCCCTGCCTATCTGTATTC-3′; reverse, 5′-TCCCCACAGGGAGTGTGTAG-3′).

For measuring proliferation of infected cells, cells were assayed at 4 weeks post infection by resazurin viability assay. Briefly, infected cells were plated on 96-well plates in triplicates with at a density of 5,000 cells per well. The blank medium was used as negative control. After culture for 48 h, resazurin was added into each well at a final concentration of 50 μM to measure cell proliferation. The reads were performed at 6 and 24 h after adding resazurin by the use of Envision 2,104 Multilabel Reader (Perkin Elmer). The relative cell survival was calculated as percentage relative to the reads from wt EBV infection from at least three independent infection experiments.

For colony formation assay, about 1 × 10^3^ infected cells were plated on 96-well plates at 1 week post infection and cultured for another 2 weeks. Colony images were acquired and processed using a Nikon TE2000-U Inverted microscope with 4 × objective, Nikon DS-Ri1 digital camera and NIS-Elements D software. Statistical analysis was performed by two-tailed Student's *t*-test.

### Data availability

Atomic coordinates and structure factors of the BNRF1 DID—DAXX HBD—H3.3-H4 complex have been deposited in the Protein Data Bank (PDB) under primary accession code 5KDM. The authors declare that all other data supporting the findings of this study are available within the article and its Supplementary Information file.

## Additional information

**How to cite this article:** Huang, H. *et al*. Structural basis underlying viral hijacking of a histone chaperone complex. *Nat. Commun.* 7:12707 doi: 10.1038/ncomms12707 (2016).

## Supplementary Material

Supplementary Information Supplementary Figures 1-7

## Figures and Tables

**Table 1:** Data collection and refinement statistics.

	**BNRF1 DID—DAXX HBD—H3.3-H4 complex**
*Data collection*	
Space group	P32 2 1
Cell dimensions	
*a*, *b*, *c* (Å)	161.2, 161.2, 117.8
*α*, *β*, *γ* (°)	90, 90, 120
Resolution (Å)	50–3.60 (3.73–3.60)*
*R*_pim_ (%)	7.3 (93.8)
*I*/σ*I*	14.6 (0.9)
Completeness (%)	99.2 (99.1)
Redundancy	11.0 (10.7)
*Refinement*	
No. reflections (total/unique)	226,960/20,715
*R*_work_/*R*_free_ (%)	23.4/28.0
No. atoms	
Protein	4,341
B-factors	
Protein	130.2
R.m.s deviations	
Bond lengths (Å)	0.013
Bond angles (°)	1.68

r.m.s., root mean square.

^*^Highest resolution shell is shown in parenthesis. One crystal was used for the data.
