# Supplementary material for: Structural basis underlying viral hijacking of a histone chaperone complex
Source: Nat Commun. 2016 Sep 1;7:12707. doi: 10.1038/ncomms12707 (PMC5025803; doi:10.1038/ncomms12707)
Supplement: Supplementary Information — Supplementary Figures 1-7 [file ncomms12707-s1.pdf]

Supplementary Figure 1

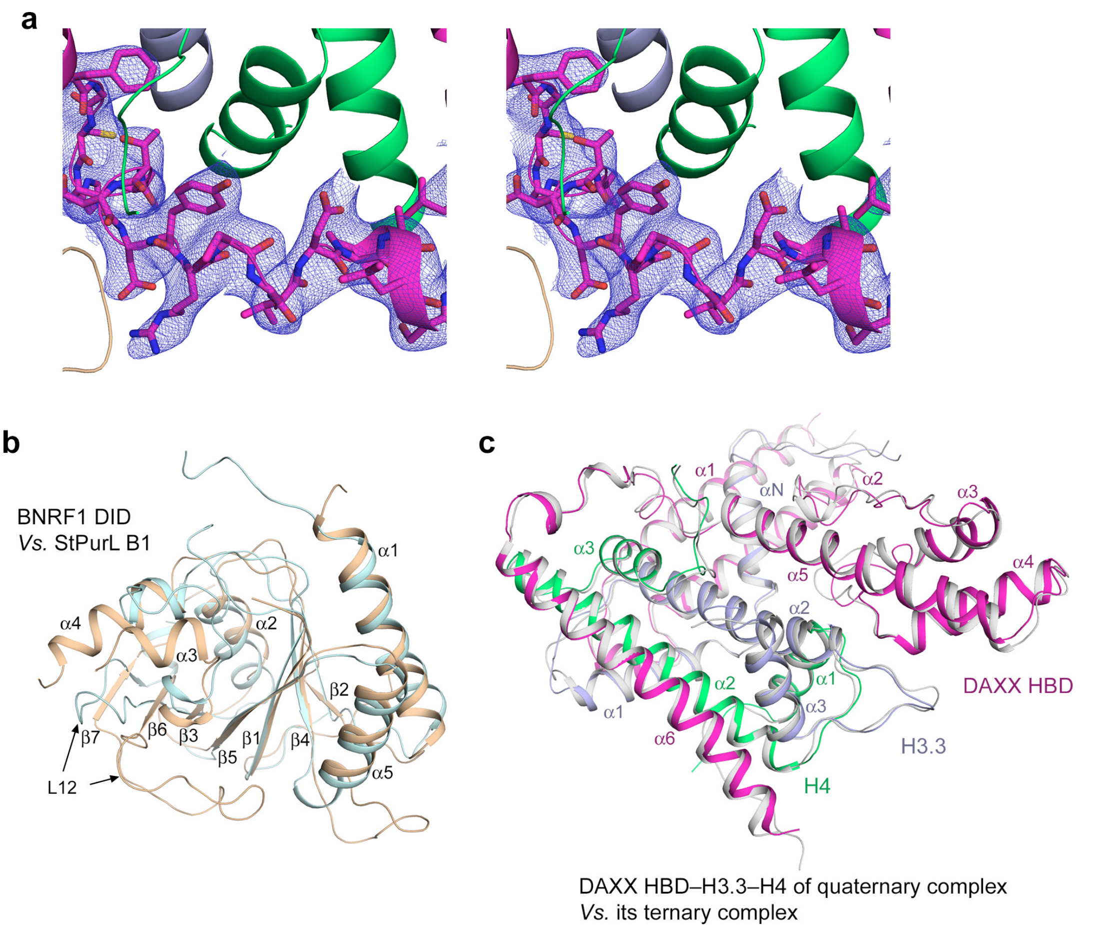

Supplementary Fig. 1. Structural Comparisons of BNRF1 DID - DAXX HBD - H3.3-H4 Complex with Related Complexes. (a) Stereoview of a portion of the final electron density map (2Fo – Fc) with a contour level of 1σ. **(b)** Comparison of the structure of the BNRF1 DID (in wheat) in the quaternary complex with the structure of the StPurL B1 lobe (derived from PDB 1T3T) (in light cyan). **(c)** Comparison of the structure of the DAXX HBD - H3.3-H4 portion in the quaternary complex in the presence of BRNF1 DID (in colors) with previous structure of this chaperone-histone ternary complex in the absence of BRNF1 DID (PDB 4H9N) (in silver).

Supplementary Figure 2

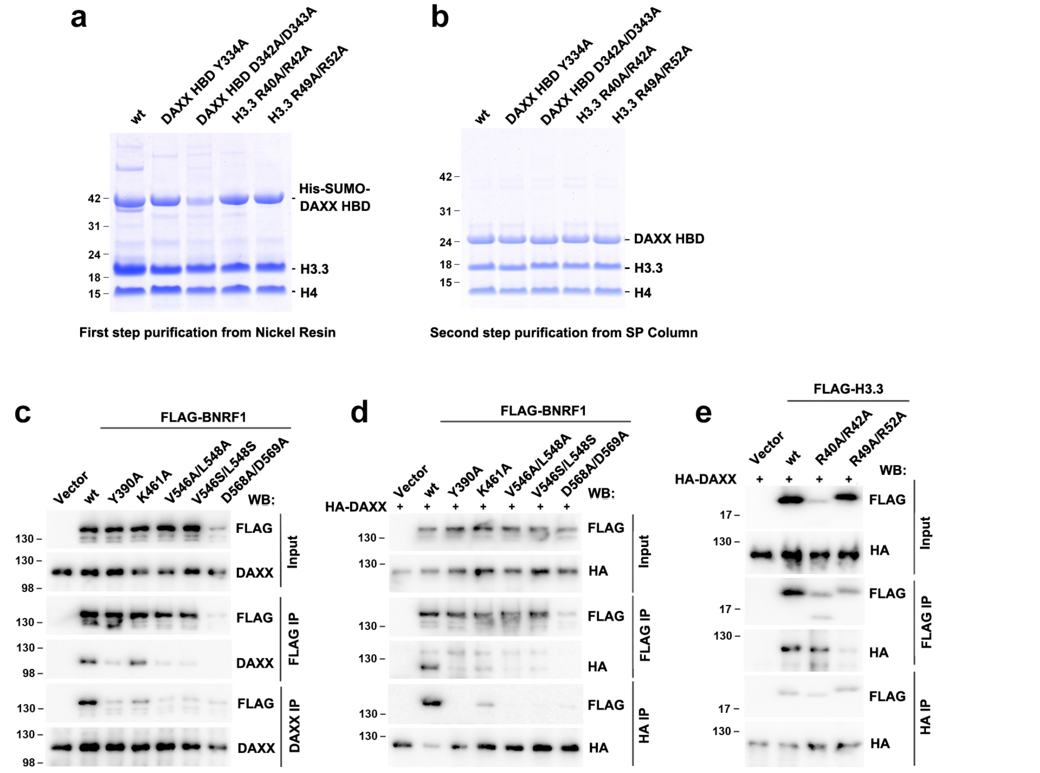

Supplementary Fig. 2. Impact of interfacial mutants on quaternary complex formation in living cells. **(a)** The SDS-PAGE gels and Coomassie staining of the mutants of the DAXX HBD-H3.3-H4 complex after purification on a nickel-affinity column. **(b)** Same as in a, except an ion exchange SP column was used for the second step purification. **(c)** FLAG-vector or FLAG-BNRF1 wt, Y390A, K461A, V546A/L548A, V546S/L548S, D568A/D569A were transfected into 293HEK cells, subject to either FLAG IP or DAXX IP, and probed with antibody to either FLAG or DAXX as indicated. Input (10%) is shown in top two panels, as indicated. **(d)** Same as in panel c, except HA-DAXX was cotransfected and HA antibody was used for IP and Western for DAXX detection. **(e)** HA-DAXX was cotransfected with FLAG-vector or FLAG-H3.3 wt, R40A/R43A, or R49A/52A and subject to FLAG-IP or HA-IP followed by Western blot with either FLAG or HA, as indicated. Input (10%) is shown in top two panels, as indicated.

Supplementary Figure 3

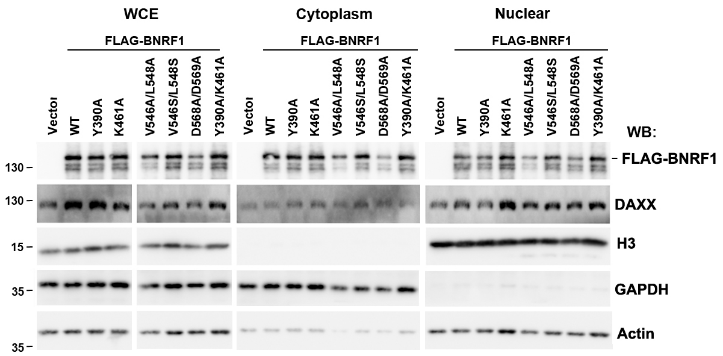

Supplementary Fig. 3. Subcellular distribution of BNRF1 wt and mutants. FLAG-vector, or FLAG-BNRF1 WT, Y390A, K461A, V546A/L548A, V546S/L548S, D568A/D569A and Y390A/K461A were transfected in Hep2 cells for 48 hrs, subject to fractionation into whole cell extracts (WCE) (left), cytoplasm (middle), or nuclear fractions (right), and then assayed by Western blot with antibodies to FLAG, DAXX, H3, GAPDH, or Actin, as indicated.

Supplementary Figure 4

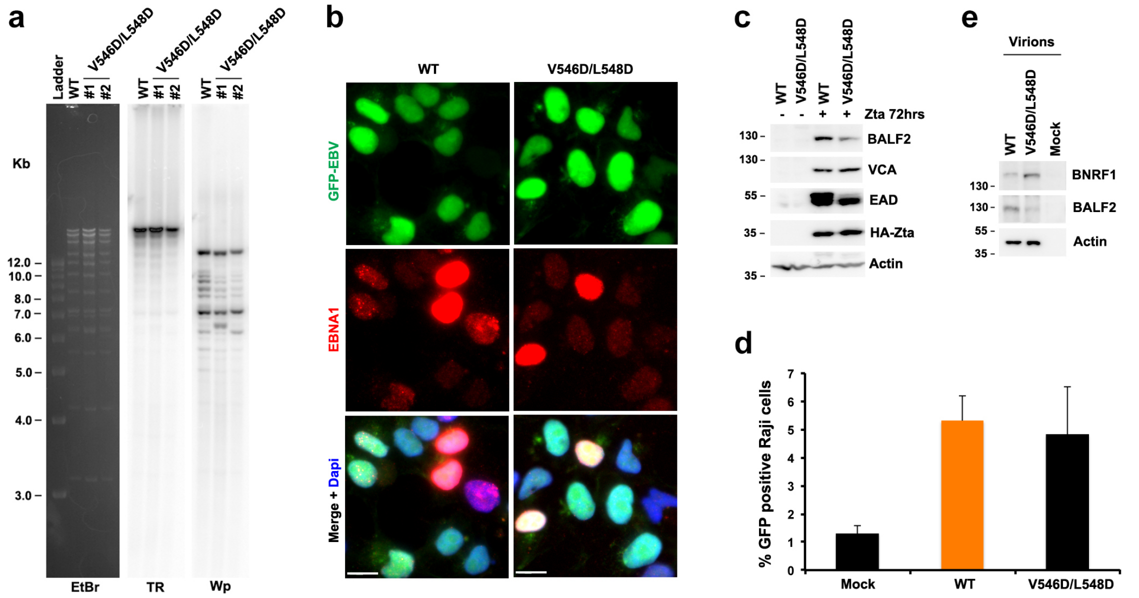

Supplementary Fig. 4. Characterization of the recombinant EBV-GFP BNRF1 V546D/D548D bacmids and viruses. (a)BACEBV-GFPwt and BNRF1V546D/L548D mutant was digested with *Eco*RI, fractionated on 0.8% agarose gel, and stained with Ethidium bromide (EtBr) followed by Southern blots with ^{32}P-labeled probes specific for Wp or TR regions. BACEBV-GFP BNRF1V546D/L548D #2 was used to generate mutant virus production cells. **(b)** Immunofluorescence analysis for EBNA1 (red) in BACEBV-GFPwt and BNRF1V546D/L548D HEK-293T virus production cell lines. GFP expression levels (green) were monitored and Dapi (blue) was shown in Merge image. Scale bar = 10 μm. **(c)** Virus production cells shown in b were either mock transfected or co-transfected with expression vectors for HA-tagged Zta and BALF4 for 72 hrs. Viral reactivation was examined by western blot with BALF2, VCA, EAD, HA, or Actin antibody, as indicated. **(d)** Raji cells were mock treated or superinfected with recombinant EBV wt (orange) or BNRF1V546D/L548D genomes at a MOI of 30 for 4 days in the presence of 100 ng ml^{-1} TPA and then assayed for Raji cell infection by FACS analysis of GFP-positive cells. The bar graph represents means ± s.d. (n=3). **(e)** Virions from HEK-293T mock cells or recombinant wt or BNRF1V546D/L548D virus production cells were assayed by western blot with antibodies to BNRF1, BALF2, or Actin.

Supplementary Figure 5

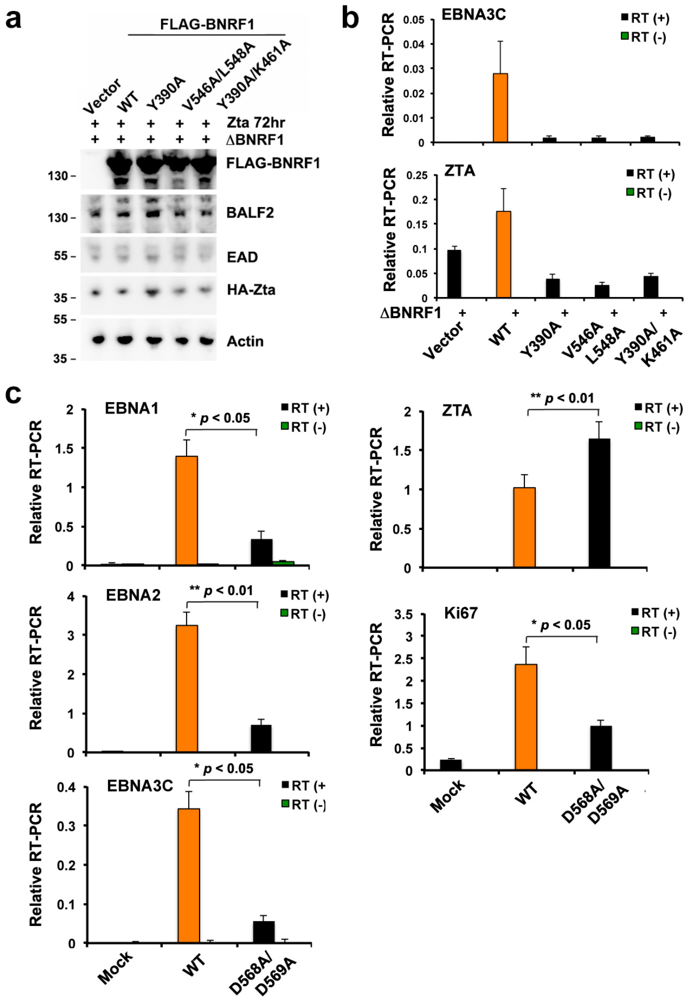

Supplementary Fig. 5. BNRF1-DAXX interaction is essential for EBV latent cycle gene expression at early stage of viral infection. (a) Virus production cells for the BNRF1 deletion mutant (ΔBNRF1) were co-transfected with expression vectors for HA-tagged Zta and BALF4 together with either empty vector, FLAG-BNRF1 wt, Y390A, V546A/L548A, or Y390A/K461A. Viral reactivation was examined 72 hrs post-transfection by western blot with FLAG, BALF2, EAD, HA, or Actin antibody, as indicated. **(b)** RT-qPCR analysis of EBNA3C and ZTA expression in B-cells infected with recombinant EBV virus generated by trans-complementation of the BNRF1 deletion mutant (ΔBNRF1), as described in Fig. 6f. **(c)** Primary human B-cells were mock treated or infected with recombinant EBV wt (orange) or BNRF1 D568A/D569A

genomes at a MOI of 30 for 72hrs and then assayed by RT-PCR for EBV latency associated genes EBNA1, EBNA2, EBNA3C, or lytic immediate ealy gene ZTA, or cellular proliferation marker Ki67. The bar graph represent means ± s.d. (n=3), two-tailed t test. No RT controls shown in green.

Supplementary Figure 6

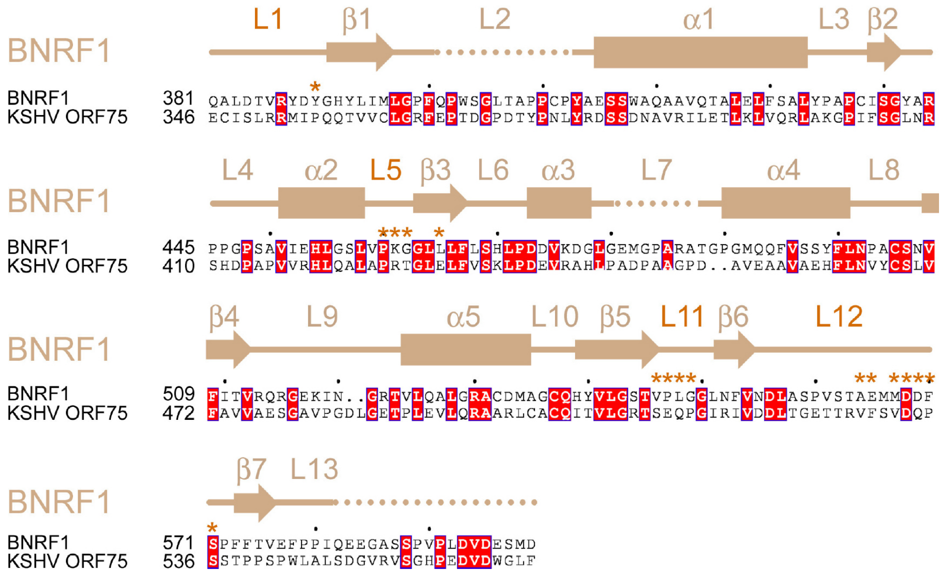

Supplementary Fig. 6. Sequence alignment of BNRF1 DID with the corresponding region of KSHV ORF75. EBV BNRF1 and KSHV ORF75 are homologous proteins. The residues in the L1, L5, L11 and L12 loops of BNRF1 DID that are important for binding to the DAXX HBD-H3.3-H4 complex are denoted with an orange asterisk. It is clear that these residues are largely not conserved between BNRF1 and KSHV ORF75.

Supplementary Figure 7

Fig. 2c

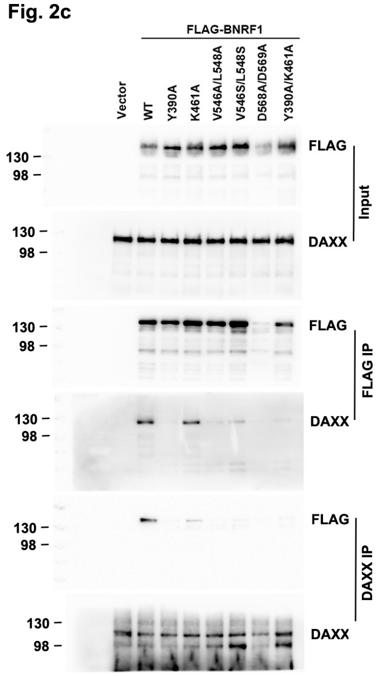

Fig. 2d

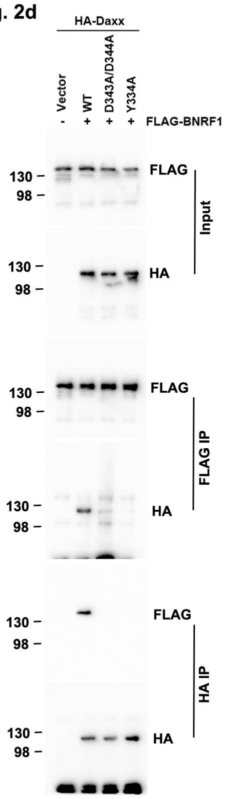

Fig. 2e

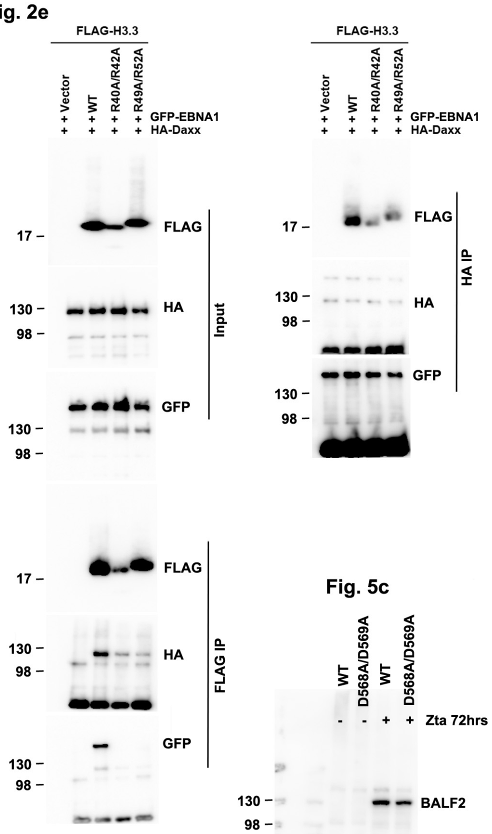

Fig. 5a

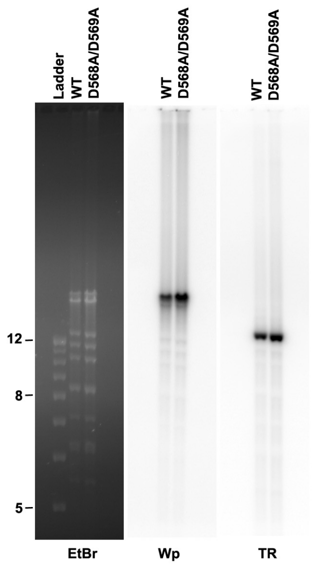

Fig. 5d

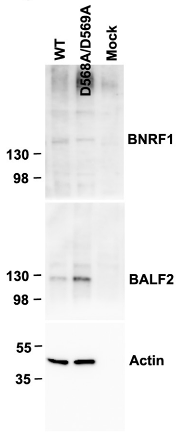

Fig. 6g

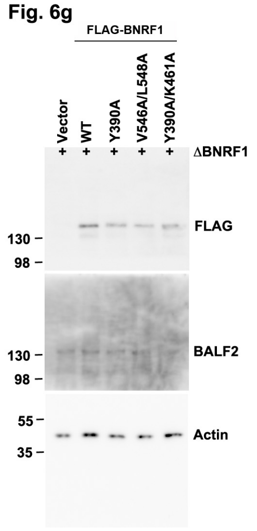

Fig. 5c

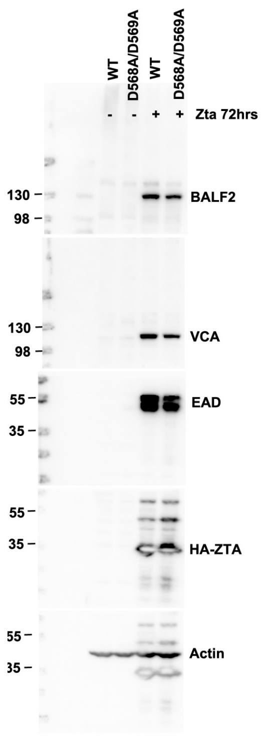

Sup. Fig. 2c

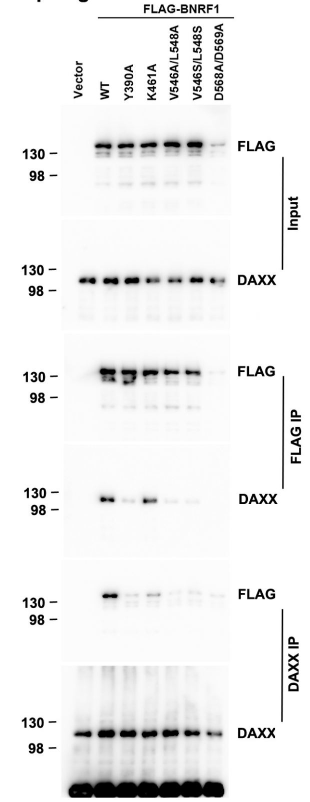

Sup. Fig. 2d

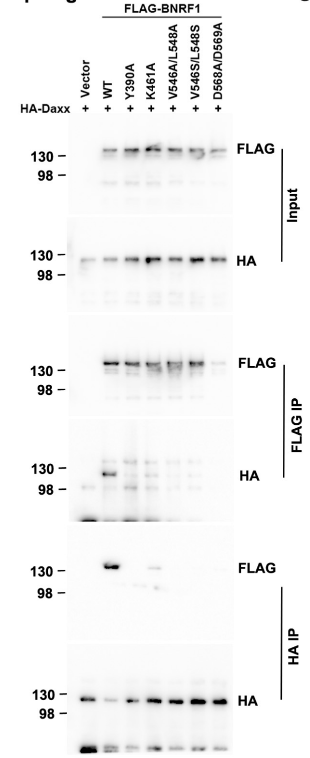

Sup. Fig. 2e

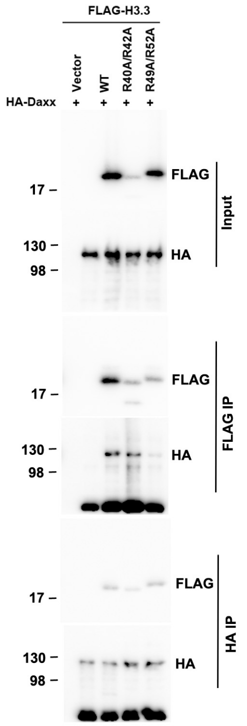

Sup. Fig. 4c

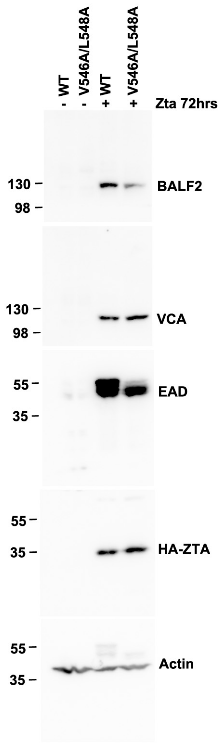

Sup. Fig. 3

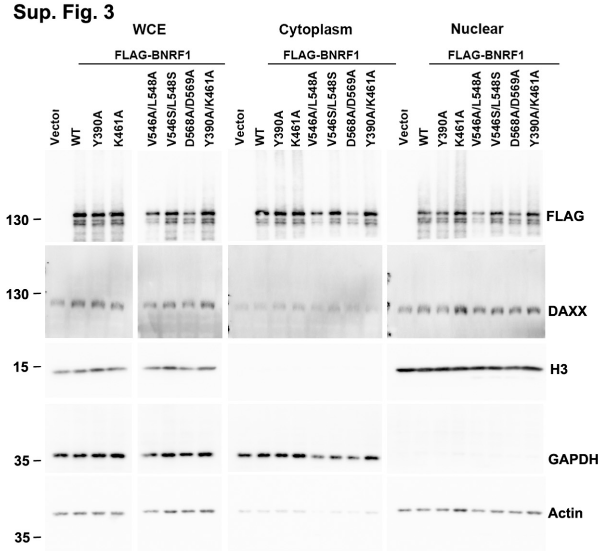

Sup. Fig. 4a

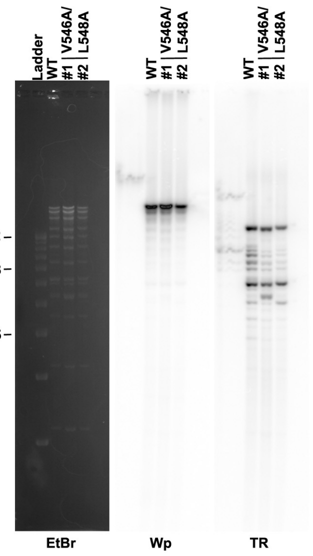

Sup. Fig. 4d

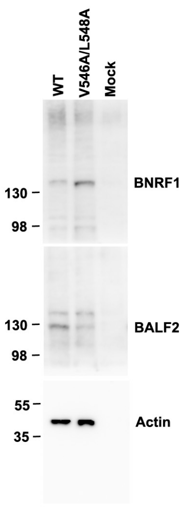

Sup. Fig. 5b

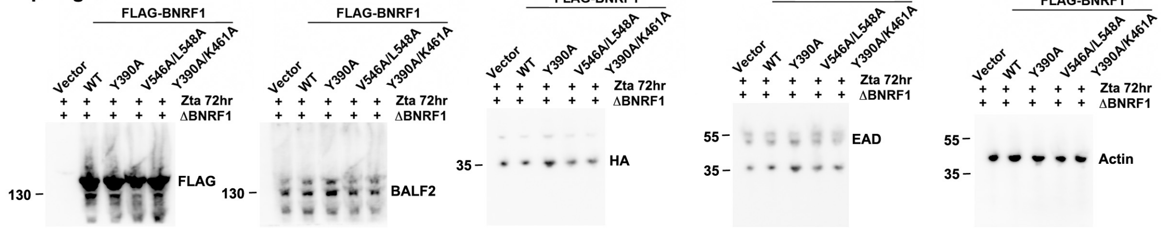

Fig. 3a

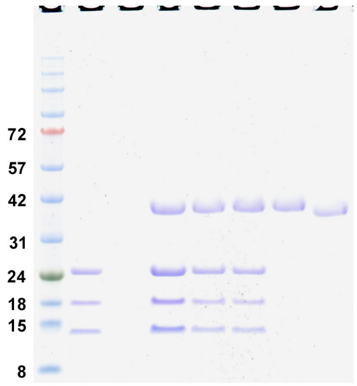

Fig. 3b

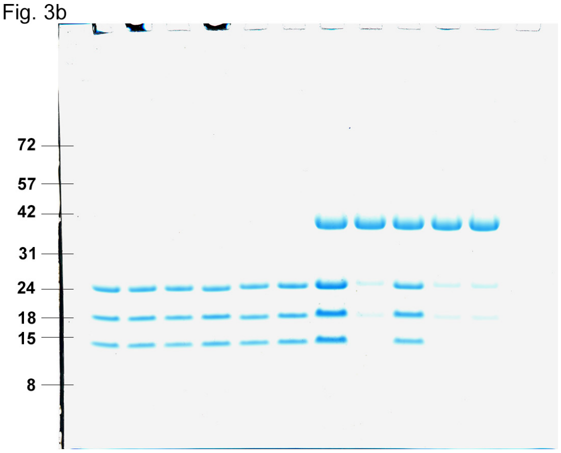

Sup. Fig. 2a

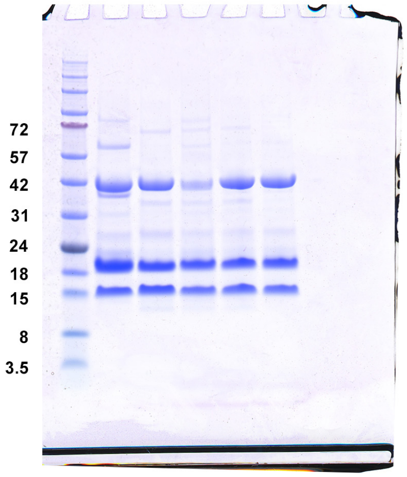

Sup. Fig. 2b

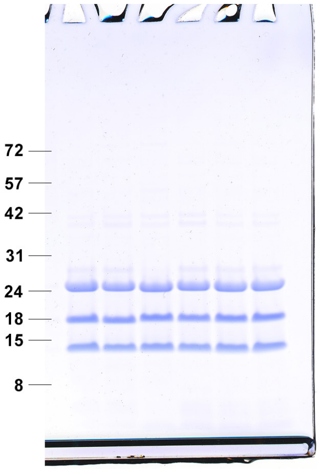

Supplementary Fig. 7. Original images of gels and western blots shown in this study.
